# Combining Generalized Renewal Processes with Non-Extensive Entropy-Based q-Distributions for Reliability Applications

**DOI:** 10.3390/e20040223

**Published:** 2018-03-25

**Authors:** Isis Didier Lins, Márcio das Chagas Moura, Enrique López Droguett, Thaís Lima Corrêa

**Affiliations:** 1 Center for Risk Analysis and Environmental Modeling—CEERMA, Universidade Federal de Pernambuco, Recife PE 50740-550, Brazil; 2 Department of Production Engineering, Universidade Federal de Pernambuco, Recife PE 50740-550, Brazil; 3 Department of Mechanical Engineering, University of Chile, Beauchef 850, Santiago, Chile; 4 Department of Mechanical Engineering, University of Maryland, College Park, MD 20742, USA

**Keywords:** generalized renewal process, q-Exponential distribution, q-Weibull distribution, particle swarm optimization, reliability analysis

## Abstract

The Generalized Renewal Process (GRP) is a probabilistic model for repairable systems that can represent the usual states of a system after a repair: as new, as old, or in a condition between new and old. It is often coupled with the Weibull distribution, widely used in the reliability context. In this paper, we develop novel GRP models based on probability distributions that stem from the Tsallis’ non-extensive entropy, namely the q-Exponential and the q-Weibull distributions. The q-Exponential and Weibull distributions can model decreasing, constant or increasing failure intensity functions. However, the power law behavior of the q-Exponential probability density function for specific parameter values is an advantage over the Weibull distribution when adjusting data containing extreme values. The q-Weibull probability distribution, in turn, can also fit data with bathtub-shaped or unimodal failure intensities in addition to the behaviors already mentioned. Therefore, the q-Exponential-GRP is an alternative for the Weibull-GRP model and the q-Weibull-GRP generalizes both. The method of maximum likelihood is used for their parameters’ estimation by means of a particle swarm optimization algorithm, and Monte Carlo simulations are performed for the sake of validation. The proposed models and algorithms are applied to examples involving reliability-related data of complex systems and the obtained results suggest GRP plus q-distributions are promising techniques for the analyses of repairable systems.

## 1. Introduction

There are different point processes that can be used to model repairable systems, which are related to important notions of reliability analysis such as repairs, spare stocks, optimal preventive maintenance and availability [1]. In all of them, failure occurrences are thought of as points in the time axis and repair times are supposed negligible when compared to operational times [2,3]. 

A repairable system can be characterized as being restorable with no need of complete replacement. It usually reaches one of the following states after a repair: “as new”, “as old” or “between new and old”. If the maintenance crew executes a perfect repair, the system’s failure intensity is restored to its initial condition as if the system were new; if the repair is minimal, the system’s failure intensity remains unaltered since last failure; otherwise, if the repair is imperfect, the system’s failure intensity is resumed to an intermediate condition between initial and since last failure.

Renewal processes (RP) and non-homogeneous Poisson processes (NHPP) are generally used to account for the states as new and as old, respectively. Nevertheless, these states are often exceptions rather than rule, from the standpoint of practical reliability engineering [4]. In this context, the generalized renewal process (GRP) [5] emerges as an alternative, since it can attend all the post-repair states due to the inclusion of the parameter of repair effectiveness, which is denoted in this work as *r*, and represents the post-repair states through the notion of virtual age [6]. Kijima [7] proposed two types of virtual age models, one that compensates only the damage accumulated during the last time between failures—type I; and the other that considers the damages since the beginning of system’s operation—type II. The application of GRP permits the estimation of reliability and maintainability of repairable systems, atmospheric phenomena and various probabilistic processes [8]. GRP has been applied with times to failure assumed to be Weibull random variables [4,8,9,10,11].

In this paper, we propose the coupling of GRP based on virtual age type I with q-distributions that come from the non-extensive Tsallis’ entropy [12,13]. Specifically, the q-Exponential and q-Weibull distributions are combined to the GRP. The former distribution is directly derived from the maximization of Tsallis’ non-extensive entropy, which is a generalization of the Boltzmann-Gibbs-Shannon (BGS) entropy, as the latter can be recovered when q → 1. The BGS entropy is adequate when different states of the system under analysis are independent and, in this case, the energy and entropy are extensive quantities. Nevertheless, some systems present a complex behavior with spatial and temporal interaction, thus the independence assumption becomes invalid. In the reliability context, when multiple failure causes compete or cooperate over time, a complex behavior can emerge and probabilistic models based on the non-extensive formalism can be adopted [14].

The q-Weibull is a probability distribution that smoothly interpolates the q-Exponential and the Weibull distributions to generate a unified framework to accommodate different cases of data adjustment [15]. Indeed, the q-Weibull distribution has the Exponential, Weibull and q-Exponential distributions as special cases and the q-Exponential generalizes the Exponential probability model. 

The q-Exponential and Weibull distributions can handle monotonically decreasing, constant and monotonically increasing failure intensities. Besides these three behaviors, the q-Weibull distribution can model two additional ones with a single set of parameters: unimodal and U-shaped (bathtub curve) [14]. In this way, the q-Exponential is an alternative to the Weibull in terms of flexibility of failure intensity modeling. Moreover, when the shape parameter (q) of the q-Exponential distribution assumes values in the interval (1, 2), it presents a power law behavior [16] that enables better data adjustment in the presence of extremely large values when compared to the Weibull model [17].

In this way, these abilities of the considered q-distributions, when combined to GRP, would enable the modelling and analyses of repairable systems according to more realistic conditions and allow decisions about reliability, maintenance planning and evaluation to be performed in a more accurate way. We also highlight that the q-Weibull-GRP is a generalization of both the widely used Weibull-GRP and the q-Exponential-GRP developed in this work and that the q-Exponential-GRP is an alternative to the Weibull-GRP. 

The parameters of the proposed models are here estimated by the maximum likelihood (ML) method due to the good statistical properties of the resulting estimators [18]: they are approximately unbiased, their limit variance is nearly as small as the variance resulting from other estimators. However, the ML framework, when applied to the q-Exponential-GRP and the q-Weibull-GRP, results in log-likelihood functions that present discontinuities for some parameters’ values and in intricate systems of first derivatives. Thus, the corresponding estimators are very difficult to be analytically obtained. Due to the complicated first derivatives, along with constraints over parameters’ values to guarantee the models’ probabilistic validity, derivative-based optimization methods may fail. Alternatively, derivative-free and nature-based heuristics (e.g., artificial bee colony (ABC) [19,20], particle swarm optimization (PSO) [21,22], among others) can be used in the quest for proper parameters’ ML estimates. The search procedure associated with these heuristics are governed by direct evaluations of the objective function, as derivative information is not required.

In this work, the chosen estimation procedure is to maximize the log-likelihood functions of the proposed q-Exponential-GRP and q-Weibull-GRP models by means of a PSO algorithm, given the above-mentioned characteristics of the related ML estimation problem and due its ease of implementation when compared to other nature-based heuristics. Additionally, PSO was originally devised to handle nonlinear optimization problems and can be used to solve problems where correlations between model parameters are high, sensitivity of the objective function to model parameters is low and the objective function is discontinuous [23,24]. It has been successfully used in different contexts. For example, [25,26,27] apply PSO in the adjustment of the hyperparameters that emerge in the training problem of support vector machines (SVM) and for feature extraction/variable selection. Ramadan et al. [28] use PSO to solve allocation problems in distribution systems with wind turbine generators. In the specific context of parameter estimation, PSO has been successfully used to estimate the parameters of mathematical models related to chemical industry [24,29,30], to obtain ML estimates of a mixture of two Weibull parameters [31], and to derive the parameters’ estimates of a Weibull model related to wind speed data from the Northeast of Brazil [32].

The estimated q-Exponential-GRP and q-Weibull-GRP models obtained by means of the PSO algorithm are then validated with a Monte Carlo simulation to obtain the expected cumulative number of failures. The proposed methods are applied to two examples involving complex systems (an angiograph and a thermal power plant), and the obtained results are also compared to the ones provided by Weibull-GRP. 

This paper unfolds as follows: Section 2 characterizes the q-Exponential and the q-Weibull probability distributions. In Section 3, GRP is briefly introduced, and the proposed q-Weibull-GRP is developed. Also in Section 3, the-q-Exponential-GRP is derived as a special case of the q-Weibull-GRP. Section 4 describes the PSO-based method for the point ML estimation of q-Weibull-GRP and q-Exponential-GRP parameters. Section 5 has the Monte Carlo procedure for validation purposes and summarizes the proposed method (parameter estimation and model validation). The application of the algorithms to reliability-related data is in Section 6. Section 7 presents the work’s conclusions. 

## 2. q-Weibull and q-Exponential Probability Distributions

Let X be the random variable related to the interval between failures and T be the random variable concerning the failure times with x and t as their respective realizations. The probability density function of the q-Weibull distribution is written as follows:

(1) $$f_{q}\left(x\right)=\left(2-q\right)\frac{\beta }{\eta }{\left(\frac{x}{\eta }\right)}^{\beta -1}\exp_{q}\left[-{\left(\frac{x}{\eta }\right)}^{\beta }\right],x\geq 0,$$

where q along with *β* are the shape parameters, while *η* is the scale parameter. Also, *q* < 2, *η* > 0 and *β* > 0. The q-Exponential function, $\exp_{q}\left(\cdot \right)$ in Equation (1), is defined as:

(2) $$\exp_{q}\left(x\right)=\{\begin{array}{c} {\left[1+\left(1-q\right)x\right]}^{\frac{1}{1-q}},\;\;\mathrm{if}\;\left[1+\left(1-q\right)x\right]\geq 0\\ \;\;\;\;\;\;\;\;\;\;\;0,\;\;\mathrm{otherwise}. \end{array}$$

Thus, replacing the q-Exponential function by its definition, Equation (1) becomes:

(3) $$f_{q}\left(x\right)=\left(2-q\right)\frac{\beta }{\eta }{\left(\frac{x}{\eta }\right)}^{\beta -1}{\left[1-\left(1-q\right){\left(\frac{x}{\eta }\right)}^{\beta }\right]}_{+}{}^{\frac{1}{1-q}},x\geq 0.$$

The support of Equation (3) changes depending on the value of the parameter q:

(4) $$x\in \{\begin{array}{c} \left[0,\infty \right),\;\;\;\;\mathrm{for}\;1<q<2,\\ \left[0,x_{\max}\right],\;\;\;\;\mathrm{for}\;q<1, \end{array}$$

where $x_{\max}=\frac{\eta }{{\left(1-q\right)}^{1/\beta }}$ is the maximum allowed time (lifetime deadline [14]) so as to preserve the probabilistic properties of Equation (3) when q<1; for these values of q, the integration of $f_{q}\left(x\right)$ diverges for $x>x_{\max}$. As mentioned before, the q-Weibull distribution has some other probability distributions as special cases: It reduces to:

- A q-Exponential distribution when *β* = 1;
- A Weibull distribution for q→1;
- An Exponential distribution for both *β* = 1 and q→1.

The q-Weibull cumulative distribution and reliability functions are given by Equations (5) and (6), respectively:

(5) $$F_{q}\left(x\right)=1-{\left[1-\left(1-q\right){\left(\frac{x}{\eta }\right)}^{\beta }\right]}_{+}{}^{\frac{2-q}{1-q}},$$

(6) $$R_{q}\left(x\right)=1-\;F_{q}\left(x\right)=\;{\left[1-\left(1-q\right){\left(\frac{x}{\eta }\right)}^{\beta }\right]}_{+}{}^{\frac{2-q}{1-q}}.$$

Both $F_{q}\left(x\right)$ and $R_{q}\left(x\right)$ are defined for the intervals shown in Equation (4), which rely on the value of the parameter q. The associated hazard rate function, defined as:

(7) $$h_{q}\left(x\right)=\frac{f_{q}\left(x\right)}{R_{q}\left(x\right)}=\frac{\left(2-q\right)\frac{\beta }{\eta^{\beta }}x^{\beta -1}}{1-\left(1-q\right){\left(\frac{x}{\eta }\right)}^{\beta }}$$

is able to represent different types of behaviors depending on the values of the shape parameters [14]:

- For 0 < β < 1 and q < 1, $h_{q}\left(x\right)$ is bathtub-shaped;
- For 0 < β < 1 and 1 < q < 2, $h_{q}\left(x\right)$ is monotone decreasing;
- For β = 1 and q → 1, $h_{q}\left(x\right)$ is constant;
- For β > 1 and q < 1, $h_{q}\left(x\right)$ is monotone increasing;
- For β > 1 and 1 < q < 2, $h_{q}\left(x\right)$ is unimodal.

As mentioned before, when the q-Weibull assumes β = 1, it reduces to the q-Exponential distribution. In this way, the corresponding PDF, CDF, reliability function and hazard rate can be obtained by setting β = 1 in Equations (3), and (5)–(7), respectively. Note that, as the q-Weibull, the q-Exponential distribution has different supports depending on the value of q; for q < 1, $x_{\max}=\frac{\eta }{1-q}$. The q-Exponential hazard rate depends only on the shape parameter q and its behaviors can be:

- Monotone decreasing for 1 < q < 2;
- Constant for q → 1; in this case, the q-Exponential reduces to an Exponential probability distribution;
- Monotone increasing for q < 1.

The hazard rate function is defined as the probability of failure given that the system has survived up to time t—a conditional probability—per unit of time. It is related only to the first failure. On the other hand, the failure intensity function ρ(t) is based on the unconditional probability of failure per unit of time and is associated to repairable systems, which are considered in GRP models. It is defined as the rate of change of the expected number of failures (E[N(t)]) with respect to time [33]:

(8) $$\rho \left(t\right)=\frac{dE\left[N\left(t\right)\right]}{dt}.$$

Based on a GRP model, an estimate of the expected number of failures by t ($E\left[\hat{N}\left(t\right)\right]$) can be obtained via Monte Carlo simulation (see Section 5). Therefore, a GRP model can be compared to the real failure occurrences in terms of the expected number of failures: $E\left[\hat{N}\left(t\right)\right]$ vs. N(t).

## 3. The Proposed q-Weibull-GRP and q-Exponential-GRP Models

The GRP can incorporate the post-repair states “as new”, “as old”, “between new and old” that a repairable system may assume. The models that have been mostly used in the reliability analysis of repairable systems are the RP and the NHPP, which can be considered particular cases of GRP [10]. However, RP and NHPP assume simplifying hypotheses, which restrict its application to realistic cases.

In contrast to the NHPP, GRP considers a conditional probability function based on the system’s virtual age, and thus the time to the next failure is conditioned to the virtual age, instead of the actual age used in NHPP. By covering major repair assumptions encountered in practice, GRP provides more flexibility in modeling real life failure occurrence processes [9]. 

GRP presents the concept of virtual age through the inclusion of the parameter r, which represents effectiveness and quality of repair. The value r = 0 leads to an RP (“as new” condition after a perfect repair), while r = 1 leads to an NHPP (“as old” condition after a minimal repair). The values 0 < r < 1 lead to an intermediate condition between “as old” and “as new” ones and is related to imperfect repair. 

Pham & Wang [34] classify the maintenance according to the degree to which the operating conditions of an item is restored. The perfect repair/maintenance is related to a system that after the event has the same lifetime distribution and failure intensity as a brand new, and generally there is a replacement of the failed item. The minimal repair/maintenance restores the system to the failure intensity it had just before it failed. The imperfect repair/maintenance is a general repair that can include the two extreme cases of minimal and perfect repairs.

The virtual age Kijima type I assumes that the ith repair can only compensate for the damage accumulated during the period between the i-th and (i−1)-th failures and it is given by:

(9) $$V_{i}=\;V_{i-1}+r\;X_{i},$$

where $V_{i}$ is the i-th virtual age, r is the parameter related to the repair effectiveness as commented above and it is supposed to be in [0, 1], $X_{i}=\;T_{i}-T_{i-1}$ is the i-th time interval between two consecutive failures with $T_{i}$ and $T_{i-1}$ as the i and (i−1)-th failure times, for i = 1, …, n and assuming $T_{0}\;=V_{0}\;=\;0$. In GRP, if $V_{i-1}\;=\;v_{i-1}$, then the time to the i-th system failure is distributed according to the following conditional CDF:

(10) $$P\left(X_{i}<x_{i}|V_{i}=\;v_{i-1}\right)=F\left(x_{i}|v_{i-1}\right)=\frac{F\left(x_{i}+v_{i-1}\right)-F\left(v_{i-1}\right)}{1-F\left(v_{i-1}\right)},$$

where F(·) is the CDF of the time to the first failure of a new (hypothetical) system. Then, Equation (10) is the conditional probability of the time to failure given that the system has survived $v_{i-1}$. Assuming a q-Weibull distribution, Equation (10) turns into:

(11) $$F\left(x_{i}|v_{i-1}\right)=1-{\left\{\frac{\left[1-\left(1-q\right){\left(\frac{x_{i}+v_{i-1}}{\alpha }\right)}^{\beta }\right]}{\left[1-\left(1-q\right){\left(\frac{v_{i-1}}{\alpha }\right)}^{\beta }\right]}\right\}}^{\frac{2-q}{1-q}}$$

and the conditional q-Weibull probability density function is:

(12) $$f\left(x_{i}|v_{i-1}\right)=\left(2-q\right)\;\frac{\beta }{\alpha^{\beta }}{\left(x_{i}\right)}^{\beta -1}{\left[1-\left(1-q\right){\left(\frac{x_{i}+v_{i-1}}{\alpha }\right)}^{\beta }\right]}^{\frac{1}{1-q}}\;{\left[1-\left(1-q\right){\left(\frac{v_{i-1}}{\alpha }\right)}^{\beta }\right]}^{\frac{q-2}{1-q}}.$$

If i = 1, Equations (11) and (12) turn into the unconditional forms of CDF and PDF since $v_{0}\;=\;0$. When Kijima virtual age type I (Equation (9)) is replaced in Equation (12), it becomes:

(13) $$f\left(x_{i}|v_{i-1}\right)=\left(2-q\right)\;\frac{\beta }{\alpha^{\beta }}\;{\left(x_{i}+r\;\sum_{j=1}^{i-1}x_{j}\;\right)}^{\beta -1}\left[1-\left(1-q\right){\left(\frac{x_{i}+r\;\sum_{j=1}^{i-1}x_{j}}{\alpha }\right)}^{\beta }\right]{\left[1-\left(1-q\right){\left(\frac{r\;\sum_{j=1}^{i-1}x_{j}}{\alpha }\right)}^{\beta }\right]}^{q-2}$$

In order to generate random numbers that follow a q-Weibull-GRP based on the virtual age type I, we can apply the inverse transform procedure [35], that is, we isolate $x_{i}$ in Equation (11) and set $F\left(x_{i}|v_{i-1}\right)=U$, where U is a uniform random number in (0,1). The resulting formula is:

(14) $$x_{i}=\alpha \;{\left\{\frac{1-{\left(1-U\right)}^{\frac{1-q}{2-q}}\left[1-\left(1-q\right)\left(\frac{r\;\sum_{j=1}^{i-1}x_{j}}{\alpha }\right)\right]}{1-q}\right\}}^{\frac{1}{\beta }}-r\sum_{j=1}^{i-1}x_{j}.$$

Equation (14) is used for validation purposes in the Monte Carlo simulation described in Section 5. The characterization of the q-Exponential-GRP based on the q-Weibull-GRP is straightforward; simply set β=1 in Equations (11)–(14).

### Maximum Likelihood Problems for the q-Weibull-GRP and the q-Exponential-GRP

We consider that failure data are available up to the time of the last failure occurrence (failure-terminated case). Considering that the first failure does not attend to the conditional probability function, then the likelihood function is given by:

(15) $$L=f\left(x_{1}\right)\;\prod_{i=2}^{n}f(x_{i}|v_{i-1}),$$

where $x_{i}$ for i = 1, …, n are the observed times between failures.

The logarithm of Equation (15) is often used in the optimization [18]; thus, the log-likelihood is the actual objective function. Moreover, some constraints must be considered to preserve the validity of the q-Weibull and q-Exponential probabilistic models: (i) non-negativity of parameters (otherwise, negative probability densities would be obtained contradicting their definition); and (ii) non-negativity of the argument of the q-Exponential function. In the development of the log-likelihood expression, these terms appear as arguments of the ln(·) function. Then, the q-Weibull-GRP likelihood problem is determined as follows:

(16) $$\begin{array}{cc} \underset{\alpha ,\beta ,q,r}{\max}\left(-n\beta \right)\ln\alpha \;+ & n\;(\ln\beta +\ln\left(2-q\right))\\ & +\;\sum_{i=1}^{n}\{\frac{1}{\left(1-q\right)}\ln\left[1-\left(1-q\right){\left(\frac{x_{i}+r\sum_{j=1}^{i-1}x_{j}}{\alpha }\right)}^{\beta }\right]\\ & +\left(\beta -1\right)\ln\left(x_{i}+r\sum_{j=1}^{i-1}x_{j}\right)\;\}+\;\sum_{i=2}^{n}\frac{\left(q-2\right)}{\left(1-q\right)}\ln\left[1-\left(1-q\right){\left(\frac{r\sum_{j=1}^{i-1}x_{j}}{\alpha }\right)}^{\beta }\right] \end{array}$$

(17) α >0,

(18) β>0,

(19) (2−q)>0, 

(20) $$\left[1-\left(1-q\right){\left(\frac{x_{i}+r\sum_{j=1}^{i-1}x_{j}}{\alpha }\right)}^{\beta }\right]>0,\;\forall i,$$

(21) $$\left[1-\left(1-q\right){\left(\frac{r\sum_{j=1}^{i-1}x_{j}}{\alpha }\right)}^{\beta }\right]>0,\;\forall i.$$

Notice that there are six logarithms in the objective function (16) and the positivity constraints (17)–(21) are related to the arguments of five of them. Only the argument of the fifth logarithmic function does not need to be explicitly considered given that $x_{i}$’s represent the observed times between failures that are always positive, and we consider r within [0, 1]. 

The usual procedure to find the maximum likelihood estimators would be to differentiate the log-likelihood function with respect to each of the parameters, make the derivatives equal to zero and solve the resulting system of equations. However, it involves intricate nonlinear equations and analytical expressions for the estimators are very difficult to be obtained. Therefore, a constrained optimization method based on PSO heuristic is adopted to solve the optimization problem (16)–(21).

The q-Exponential-GRP likelihood problem, in turn, can be derived using the PDFs in Equation (15). Alternatively, the q-Weibull-GRP likelihood problem in Equations (16)–(21) can be reduced to the q-Exponential-GRP case by setting β=1 in Equations (16), (20) and (21) and by eliminating constraint (19).

## 4. PSO for ML Estimation of the GRP Parameters

PSO is based on the social behavior of biological organisms and has as a basic element a particle that can fly throughout the search space of the problem toward an optimum using its own information and the information provided by other particles within its neighborhood [21]. In PSO, each particle is a potential solution and the swarm evolution is based on a pair of update equations that define particles’ velocities and positions according to social and individual information about the most prominent locations. In the following, we describe the PSO for the more general q-Weibull-GRP case. For the q-Exponential-GRP, one has to remove parameter β or set its range of definition as [1, 1] (with length 0, as the q-Weibull-GRP reduces to the q-Exponential-GRP when β=1) and use the corresponding q-Exponential-GRP log-likelihood as objective function.

For the q-Weibull-GRP, the objective function used in the PSO algorithm is the q-Weibull GRP log-likelihood function. It has to be maximized by choosing optimal parameters’ estimates $\hat{\alpha },\;\hat{\beta },\hat{q},\;\hat{r}$. Then, the considered problem involves a four-dimensional search space, where each dimension is related to each of the decision variables α, β, q and r. Therefore, a particle $j=1,\;\ldots ,\;n_{part}$ has current position ($s_{j}$), the best position it has visited ($p_{j}$) and velocity ($v_{j}$) as four-dimensional vectors that have their entries associated with α, β, q and r.

**Figure 1:**
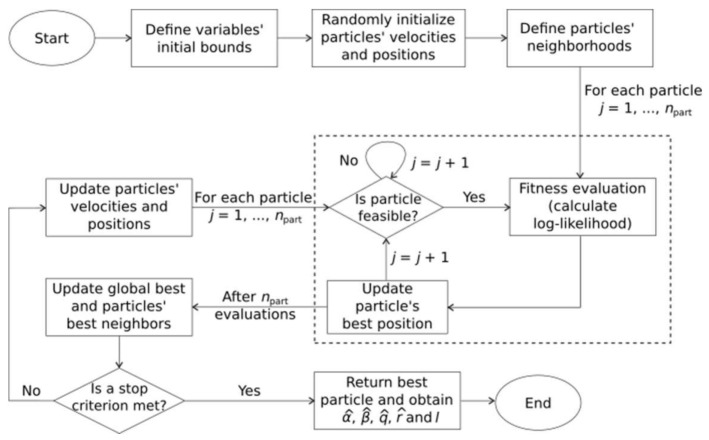
Flowchart of the PSO algorithm for the more general q-Weibull-GRP maximum log-likelihood problem. For the q-Exponential-GRP, either eliminate parameter β or set its range of definition as [1, 1].

The steps of the PSO algorithm are summarized in Figure 1. The particles’ initialization procedure involves the random generation of particles’ positions within ranges defined in the previous step (“Define variables’ initial bounds”, Figure 1). However, some combinations of values for α, β, q and r may result in infeasible particles with respect to the constraints related to the maximum log-likelihood problem considered. In the initialization step, whenever an infeasible position is generated it is immediately discarded, and a new position is then created. This stage ends when all particles’ positions are feasible in the initial swarm. 

The maximum velocities are defined as a fraction of 0.1 of the position limits of the parameters and the velocities are obtained from uniform random numbers in the maximum velocities interval defined. The positions and velocities are obtained from uniform distributions over the intervals of definition of the decisions variables and by setting a maximum velocity value $v_{\max}$, respectively. 

The particles’ neighbors are defined considering the particles’ generation order and not taking into account any sort of distance metrics. The particle i has i−1 and i+1 as neighbors. If i = 1, then the “left” neighbor is the last particle and, conversely, if the last particle is considered, its “right” neighbor is the first particle. 

After the definition of particles’ neighbors, the fitness evaluation step takes place. It consists in evaluating the log-likelihood function for each particle’s position. Then, each particle has its own best position updated if the current position returns a better log-likelihood value. After the entire swarm is evaluated, the best neighbors and global best are updated. If none of the stop criteria is met, new velocities and positions are calculated through the following equations:

(22) $$v_{jk}\left(it+1\right)=\;\chi \left\{v_{jk}\left(it\right)+\;c_{1}u_{1\;}\left[p_{jk}\left(it\right)-s_{jk}\left(it\right)\right]+c_{2}u_{2\;}\left[p_{gk}\left(it\right)-s_{jk}\right]\right\},$$

(23) $$s_{jk}\left(it+1\right)=\;s_{jk}\left(it\right)+\;v_{jk}\left(it+1\right),$$

where it is the iteration number, χ is the constriction factor that avoids velocity explosion during iterations, $c_{1}$ and $c_{2}$ are positive constants, $u_{1\;}$and $u_{2\;}$are independent uniform random numbers between 0 and 1, $p_{gk}$ is the k-th entry of vector $p_{g}$ related to the best position that has been found by any neighbor of particle j. The fitness evaluation step is repeated only for feasible particles (“let particles fly” strategy). This procedure is repeated until a stop criterion is met. The algorithm’s output of the best particle are the ML estimates and the log-likelihood value. For further details, see [21,36].

Since PSO is a probabilistic heuristic, it may provide different results for the same problem in different runs. Thus, it may be replicated several times to assess its ability in finding essentially the same parameter estimates. Usually, a unique solution is found, but depending on the characteristics of the objective function being considered, it may provide different results (local optima). Therefore, the validation of the estimated GRP models is crucial, and it is described in the next section.

## 5. Estimation of the Expected Number of Failures via Monte Carlo Simulation

In order to validate the GRP parameter estimates obtained via PSO, a Monte Carlo algorithm can be used to compare simulated outcomes with real failure data. Specifically, based on the GRP parameter estimates, the simulation returns the expected number of failures up to time $t_{i}$, which is confronted with the real number of failures by $t_{i}$, with i = 1,…,n. The simulation procedure used in this work to validate q-Exponential and q-Weibull GRPs is similar to the one used by [10] with Weibull-based GRP models.

**Figure 2:**
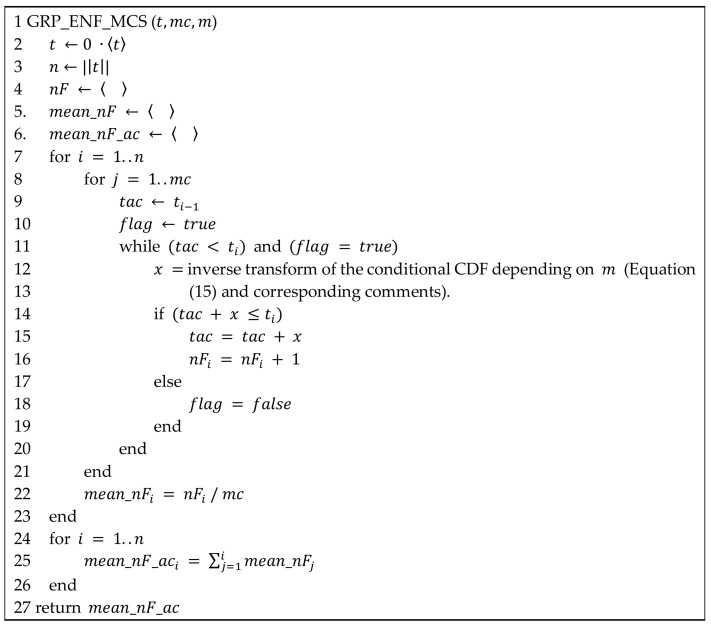
Pseudocode of the Monte Carlo simulation to obtain the expected number of failures for each observed interval $\left(t_{i-1},t_{i}\right)$, i = 1, …, n, using an estimated GRP model for validation purposes.

The pseudocode of the algorithm to obtain the GRP expected number of failures via Monte Carlo simulation (GRP_ENF_MCS) is shown in Figure 2. The inputs are the vector of observed failure times (t), the number of Monte Carlo replications (mc) and the GRP model (m= q-Exponential-GRP or q-Weibull-GRP). Lines 2–6 of the pseudocode correspond to the insertion of 0 into vector t to include $t_{0}\;=\;0$, the definition of n as the length of vector t (including the 0-th observation), the initialization of the vectors nF, mean_nF and mean_nF_ac that will contain the total number of failures in each of the intervals $\left(t_{i-1},t_{i}\right)$, the mean number of failures in $\left(t_{i-1},t_{i}\right)$ and the cumulative mean number of failures up to $t_{i}$, with i = 1, 2, …, n, respectively. Then, for each interval $\left(t_{i-1},t_{i}\right)$, mc replications are executed to obtain the corresponding mean number of failures (lines 8–22). 

Therein, the variable tac is the observed time of failure i−1 and x is the additional time for the occurrence of the i-th failure; x is obtained by the inverse transform in Equation (15) using the parameter estimates of the analyzed GRP model (lines 12–13). If tac + x is lower than or equal to $t_{i}$—the upper bound of the current interval $\left(t_{i-1},t_{i}\right)$—then tac and nF are updated (lines 15 and 16, in this order). Otherwise, the variable flag becomes false, line 8 is reached and another Monte Carlo replication begins. When the mc replications have been performed, the mean number of failures in $\left(t_{i-1},t_{i}\right)$ is obtained (line 22) and another subinterval should be analyzed (line 7). At the end, the cumulative mean number of failures for each interval is calculated (lines 24–26) and the algorithm should return the corresponding vector mean_nF_ac (line 27).Thus, for a given sample with failure data of the analyzed equipment, the proposed method to find the ML estimates for the GRP models based on either the q-Weibull or the q-Exponential distributions has the following steps:

1. Select a probability distribution to couple with GRP;
2. Run PSO for the maximum log-likelihood problem related to the GRP model chosen in the previous step;
3. Repeat step 2 for $n_{pso}$ times;
4. Identify groups of parameter estimates provided by PSO in step 3;
5. Validation: for each group of parameters’ estimates given in step 4, perform a Monte Carlo simulation to compare the expected cumulative number of failures provided by the estimated GRP model with real data;
6. Select the group of parameters’ estimates that best fits the real failure data.

This method can be repeated for each of the proposed GRP models (q-Weibull-GRP and q-Exponential-GRP) so as to decide which one is the most appropriate for the considered sample. Also, other distributions could be taken into account in step 1 (e.g., Weibull), but the PSO and Monte Carlo simulation algorithms should be adapted to handle other distributions.

## 6. Application Examples Involving Reliability-Related Data

In this section, the proposed q-Weibull GRP and q-Exponential GRP are applied to system failure data. Additionally, for the sake of comparison, the Weibull-GRP is also considered to model the same data sets. Specifically, two data sets are used, and they are related to: (i) an angiograph—data set #1, Section 6.1—and (ii) a thermal power plant—data set #2 [37], Section 6.2. They are related to different complex systems (equipment vs. plant) of different contexts (medical vs. electrical power grid), which suggests the flexibility of the proposed models in handling data from a wide range of practical applications.

The maximum likelihood estimates for the parameters of each GRP model were obtained by means of the PSO algorithm described in Section 4 with $n_{part}\;=\;30$, $n_{ite}=\;4000$, $n_{neigh}\;=\;2$. Particles’ positions were randomly initialized using a uniform distribution with the following intervals: [0.1, max(t)] for η, [0.1, 10] for β, [−10, 1.9] for q and [0, 1] for r. The adopted stopping criteria were: (i) the best particle has not changed for 400 consecutive iterations (10% of $n_{ite}$) or (ii) the attainment of $n_{ite}$. Indeed, only criterion (i) was actually used, and this indicates that the PSO could rapidly find optimal results. The algorithm was replicated 30 times.

For validation purposes, Monte Carlo simulations (mc = 10,000) were performed to stablish comparisons among the GRP models when challenged to real failure occurrences. For each data set and GRP model, we estimated the expected number of failures up to $t_{i}$—$E\left[\hat{N}\left(t_{i}\right)\right]$—using the maximum likelihood estimates for the GRP parameters given by PSO. Note that $t_{i}$ is an observed failure time given by the sum of the first i times between failures. The performance of the estimated models was visually inspected based on the expected and observed cumulative number of failures vs. time and by the mean absolute error (MAE) given by:

(24) $${\mathrm{MAE}}_{m}=\sum_{i=1}^{n}\frac{\left|E\left[\hat{N}\left(t_{l}\right)\right]-N\left(t_{i}\right)\right|}{n}$$

with $N\left(t_{i}\right)$ as the actual number of failures up to $t_{i}$ and m= Weibull, q-Exponential or q-Weibull.

### 6.1. Data Set #1: Angiograph

An angiograph is a technology-intensive clinical equipment used for imaging exams. With help of a contrast agent, doctors can visualize blood vessels and blood flow [38,39]. The times between failures (in days) of the considered angiograph are in Table 1 (to be read across the row). 

For data set #1, the PSO replications returned approximately the same parameter estimates for each of the GRP models, as can be observed in Table 2. The estimated Weibull-GRP and q-Exponential-GRP indicated an increasing failure intensity, while the q-Weibull-GRP resulted in a unimodal behavior. All GRPs models suggested an imperfect repair, that is, the angiograph returns to operation with an intermediate condition between “as new” and “as old” after a corrective maintenance.

**Figure 3:**
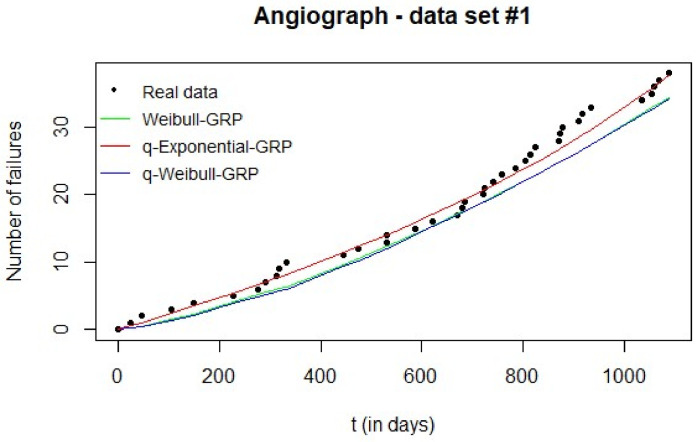
Results of the Monte Carlo simulation for data set #1: expected number of failures of each estimated GRP model can be compared to real data.

Figure 3 shows the expected number of failures given by each GRP model and the observed cumulative number of failures vs. time. The superiority of the q-Exponential-GRP is evident in the graph and is corroborated by the corresponding MAE value when compared to the other MAEs in Table 3. Additionally, the estimated Weibull-GRP and q-Weibull-GRP provided similar adjustments to the real failure data (Figure 3) with an advantage for the Weibull-GRP (smaller value of MAE when compared to the q-Weibull-GRP counterpart, see Table 3). 

### 6.2. Data Set #2: Thermal Power Plant

The times between failures, in hours, of the considered thermal power plant are shown in Table 4 [37] (to be read across the row).

For data set #2, the PSO replications returned two different solutions (named A and B) for each of the Weibull-GRP and the q-Weibull-GRP models. On the other hand, the PSO for the q-Exponential-GRP returned essentially the same parameter estimates in all 30 replications. The obtained results for data set #2 are shown in Table 5. For the Weibull-GRP and q-Weibull GRP, the corresponding columns are related to solutions A and B and the number of times out of the 30 replications in which they were found are shown in parentheses; the latter is used to calculate the descriptive statistics (mean and standard deviation—SD) of the parameter estimates. Table 5 also contains the behavior of the failure intensity and the type of repair associated with each of the solutions.

The fact that the PSO has found two solutions in different replications of the algorithm for the Weibull-GRP and q-Weibull-GRP cases is an indication of the complexity of the corresponding log-likelihood functions that govern the quest for optimal parameter estimates. Also, from Table 6, in both cases, solution A presents higher log-likelihood value and occurs less frequently when compared to solution B (−504.9050 vs. −509.8908; 7 vs. 23 and −503.7786 vs. −507.3658; 3 vs. 27 for the Weibull-GRP and q-Weibull-GRP, respectively). Then, by considering only the log-likelihood value as a criterion, we would select the respective solution A for both GRP models. 

**Figure 4:**
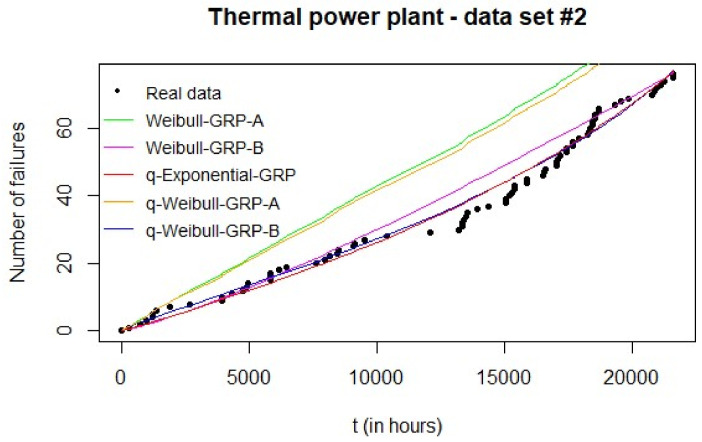
Results of the Monte Carlo simulation for data set #2: expected number of failures of each estimated GRP model can be compared to real data.

However, the validation phase with the Monte Carlo simulation results compared to the real failure data (Figure 4) clearly suggests solution B as the best if either Weibull-GRP or q-Weibull-GRP were chosen. These results are confirmed by the MAE values associated with these GRP models in Table 6—15.2202 (A) vs. 3.5504 (B) and 13.7140 (A) vs. 2.1279 (B) for Weibull-GRP and q-Weibull-GRP, in this order. A similar situation has been observed by Moura et al. [40]: the GRP parameter estimates that provided a better adjustment were not necessarily associated with the highest log-likelihood, but with a smaller error value.

Therefore, despite the lower log-likelihood value of solution B (local optimum), it is well adjusted to reality and its higher frequency within the 30 PSO replications may indicate that. This application example illustrates the importance of the validation step in the event of multiple PSO solutions: several replications of the PSO indicate the potential parameter estimates and the Monte Carlo simulation suggests which of them should be actually used. Thus, other criteria besides the log-likelihood can be considered such as MAE.

When comparing the GRP models for data set #2, the Monte Carlo simulation indicated the q-Weibull-GRP (B) as the best fit to the real failure data. This can be visually verified in Figure 4 and by the corresponding MAE in Table 6. Thus, if the several replications of the PSO algorithm provide essentially the same parameter estimates (as commonly expected), the Monte Carlo simulation is required to select which model should be chosen to represent the failure-repair behavior of the analyzed system. 

By visual inspection of Figure 4, the Weibull-GRP (B) presented the worst performance when compared to the q-Exponential-GRP and the q-Weibull-GRP (B). The last two models had similar performances towards the end of the observation period, but the q-Weibull-GRP (B) provided a better adjustment in the beginning. This result is credited to the flexibility of the q-Weibull-GRP (B) in modeling a bathtub-shaped failure intensity. The other models—Weibull-GRP (B) and q-Exponential-GRP—suggested an increasing failure intensity. In terms of type of repair, both q-Exponential-GRP and q-Weibull-GRP (B) indicated a minimal repair (system returns to an “as old condition” after a corrective maintenance). On the other hand, the estimated Weibull-GRP (B) was related to an imperfect repair. Indeed, only Weibull-GRP (B) returned an estimate between 0 and 1 for parameter r; all the other models provided extreme values (either 0 or 1). These results are summarized also in Table 5.

### 6.3. Computational Data and Further Discussion

Both PSO and Monte Carlo simulation algorithms were implemented in MATLAB R2017b and all experiments were performed on a PC with an Intel Core i7-5500U CPU @ 2.40 GHz, 8 GB RAM and Windows 10 operating system. For each application example and each GRP model, the PSO algorithm was replicated 30 times. Table 7 shows the average time, in minutes, and the mean number of iterations until convergence required by a PSO repetition per GRP model and application example. The greater effort related to the q-Exponential-GRP and the q-Weibull-GRP can be justified by the complexity of their log-likelihood functions when compared to the Weibull-GRP counterpart. Additionally, the fact that the second application needed more time than the first example can be explained by the different sample sizes related to them (data set #2 with 77 observations and data set #1 with 38 observations).

The Monte Carlo simulations to obtain the expected number of failures for validation purposes demanded low amount of time; all cases required less than 2.3 s, as shown in Table 8. Application 1 required less time than example 2 due to the smaller sample size involved in the former.

Given that the q-Weibull distribution has the q-Exponential and Weibull models as special cases, the former is expected to provide a better adjustment than the other two. However, as commented by Picoli et al. [15], there are situations in which the fit with the q-Weibull model does not give a significant improvement when compared with that obtained with the q-Exponential or Weibull distributions. In these cases, either the parameter β is close to one, indicating a q-Exponential distribution or the parameter q is close to one, leading to a Weibull model. This suggests that, for a given data set of times between failures, a fit with the q-Weibull model should be performed and then, based on β and q estimates, either a q-Exponential or a Weibull model should be considered or not. 

Nevertheless, for the two application examples considered in this work, the observation of the estimates’ values for the parameters β and q was not sufficient to decide which of the couplings q-Weibull-GRP, q-Exponential-GRP and Weibull-GRP was the most appropriate for a given case. For data set #1, the q-Weibull-GRP provided $\hat{\beta }=2.3865$ and $\hat{q}=1.2637$, which indicate that the q-Weibull-GRP would be the most suitable model. The three models gave similar log-likelihood values (with a slight advantage for the q-Weibull-GRP), but in terms of MAE evaluated by means of the expected number of failures vs. real data, the q-Exponential-GRP was the best model. For data set #2, the q-Weibull-GRP (B) returned $\hat{\beta }=0.8247$ and $\hat{q}=0.9800$, which suggest either q-Exponential-GRP or Weibull-GRP as potentially better models. But, for this example, the q-Weibull-GRP (B) was the best one both in terms of log-likelihood and MAE. 

Thus, the reasoning discussed by Picoli et al. [15] is not necessarily sufficient to determine which GRP model is the best one for a given data set. Possibly, as the GRP parameter r is included, it may in some way influence the values of the q-Weibull parameters and the assessment of their proximity to one may not be appropriate. Therefore, we recommend that the considered failure data set should be fit by each of the GRP models and then log-likelihood values along with MAE (expected number of failures vs. real data) should guide the decision of which model is the most suitable.

## 7. Conclusions

In this paper, GRP models based on virtual age Kijima type I were developed using times to failure governed by the q-Weibull and the q-Exponential probability distributions that stem from the Tsallis’ non-extensive entropy. The GRP is a more general stochastic process that generalizes RP and NHPP as it presents the parameter r related to efficacy of the corrective maintenance actions. The considered q-distributions are promising alternatives to the Weibull model, which is widely used with GRP. Thus, this paper aimed at providing alternative GRP models for the adjustment of failure data of complex systems to enable a more realistic support for maintenance-related decisions.

Specifically, the q-Weibull-GRP is more flexible than the q-Exponential-GRP and Weibull-GRP due to the presence of an additional shape parameter that enables it to model reliability-related data of systems presenting non-monotone failure intensity functions (e.g., bathtub-shaped and unimodal). In terms of failure intensity modeling, both q-Exponential-GRP and Weibull-GRP can fit data with monotone decreasing and monotone increasing failure intensities. Nevertheless, the q-distributions are expected to have superior performance when modeling data from complex systems that may have many multiple and interacting causes acting on a cooperative/conflicting basis to generate a failure.

In order to estimate the parameters of the proposed GRP models, the corresponding maximum likelihood constrained problems were developed, and they were solved by means of a PSO algorithm. The application of PSO is justified by the intricacy of the log-likelihood functions to be maximized. Also, a Monte Carlo simulation was devised to confront the estimated GRPs given by PSO with real data. 

The models and algorithms were applied to two data sets related to failures of complex systems (a thermal power plant and an angiograph). In both applications, a q-distribution presented better performance, possibly due to the interdependence of causes that may lead the considered system to a failed state. Specifically, for the angiograph, the q-Exponential-GRP provided the best adjustment (increasing failure intensity and imperfect repair), while for the thermal power plant, the q-Weibull-GRP was the best model (bathtub-shaped failure intensity and minimal repair). For both examples, the best MAE values were low, which is an indication of the suitability of the proposed models to fit the given data sets. The role of the validation phase involving Monte Carlo simulations was also discussed in the application examples when single or multiple solutions are given by the estimation procedure (PSO algorithm in this work).

As it is rather common in practice, the value of the parameter r (related to repair efficacy) was constrained to the interval [0,1] leading to situations where the system condition is restored to an intermediate state between “new” and “old”. Depending on the failure intensity considered, there are different interpretations to the repair.

Traditionally, r = 0 and r = 1 are related to perfect and minimal repairs, respectively, in the sense that if the failure intensity is monotonically increasing, then the system returns to operation “as good as new” and “as bad as old”. Probably, this might be credited to the wide application of GRP models to system in the degradation phase. However, even with the terms “perfect” and “minimal” being applied for the other failure intensity behaviors, such a reasoning is not completely appropriate. Particularly, if the failure intensity is decreasing, the idea is indeed opposite: r = 0 (perfect repair; “as new” condition) and r = 1 (minimal repair; “as old” condition) would imply bad and good situations, respectively.

Also, in this paper, a unique value was assumed for the parameter r and it may not be the same for all periods of system’s lifecycle when it is related to non-monotone failure intensities. Dijoux [41] addressed this problem with the Weibull-GRP making assumptions of repairs and using different virtual ages to each phase of the bathtub curve resulting in a complex system with an elevated number of parameters to be estimated, which is rather not viable in practice. These issues are topics of ongoing research, along with the investigation of other q-distributions to be coupled with GRP. Also, future works should consider other heuristics (e.g., ABC) as ML estimation procedures for comparison purposes with the PSO algorithm here adopted.

## Figures and Tables

**Table 1:** Data set #1: times between failures (in days)—angiograph.

24	23	57	46	78	48	16	21	6
13	112	31	55	1	55	36	48	9
5	37	2	18	18	27	18	11	8
48	3	5	30	8	17	101	19	4
11	20							

**Table 2:** Maximum likelihood results for data set #1 from 30 replications of PSO (angiograph).

		Weibull-GRP	q-Exponential-GRP	q-Weibull-GRP
$\hat{\eta }$	Mean	56.3912	47.2943	42.3529
	SD	1.8386 × 10^−6^	2.1859 × 10^−6^	4.1117 × 10^−6^
$\hat{\beta }$	Mean	1.6449	-	2.3865
	SD	2.9867 × 10^−8^	-	1.5205 × 10^−7^
$\hat{q}$	Mean	-	0.9529	1.2637
	SD	-	2.8249 × 10^−8^	5.9936 × 10^−8^
$\hat{r}$	Mean	0.0999	0.5676	0.0591
	SD	1.2011 × 10^−8^	3.3768 × 10^−7^	9.5791 × 10^−9^
l	Mean	−159.1246	−159.9368	−158.7174
	SD	6.5500 × 10^−14^	9.2800 × 10^−14^	4.2600 × 10^−14^
Behavior of failure intensity		Increasing	Increasing	Unimodal
Type of repair		Imperfect	Imperfect	Imperfect

**Table 3:** Mean absolute errors for the GRP models for data set #1; best value in bold.

	Weibull-GRP	q-Exponential-GRP	q-Weibull-GRP
MAE	2.2780	1.0218	2.4254

**Table 4:** Data set #2: times between failures (in hours)—thermal power plant [37].

275.883	10.633	455.350	205.100	279.900	6.017	117.550	546.850	767.383
1249.700	41.483	385.900	438.417	134.767	23.783	895.933	1.800	0.267
331.217	295.467	1157.283	327.883	230.217	300.483	48.317	566.083	51.583
382.783	854.883	1719.250	1098.117	151.483	4.550	52.667	122.983	32.200
401.517	432.550	673.200	10.950	121.883	128.017	81.600	14.633	463.850
21.800	626.717	13.833	71.750	448.350	8.433	19.250	113.917	217.950
36.983	203.717	1.233	262.767	324.400	42.733	56.283	112.383	0.817
74.333	10.400	132.400	30.917	621.083	223.617	315.433	895.617	130.883
79.117	154.433	124.700	363.217	14.133				

**Table 5:** Maximum likelihood results for data set #2 (thermal power plant).

		Weibull-GRP		q-Exponential-GRP	q-Weibull-GRP	
		A (7)	B (23)	(30)	A (3)	B (27)
$\hat{\eta }$	Mean	235.1716	552.1272	460.7025	593.2604	254.9368
	SD	2.1984 × 10^−6^	5.4350 × 10^−5^	1.3677 × 10^−5^	1.7961 × 10^−4^	1.4653 × 10^−4^
$\hat{\beta }$	Mean	0.7343	1.2234	-	0.5984	0.8247
	SD	1.2120 × 10^−8^	4.0990 × 10^−8^	-	5.6551 × 10^−8^	1.3825 × 10^−7^
$\hat{q}$	Mean	-	-	0.9861	0.5631	0.9800
	SD	-	-	7.4458 × 10^−10^	1.5043 × 10^−7^	5.4823 × 10^−9^
$\hat{r}$	Mean	0.0000	0.4974	1.0000	0.0000	1.0000
	SD	4.8738 × 10^−12^	2.1340 × 10^−7^	1.4750 × 10^−13^	1.3375 × 10^−11^	9.7453 × 10^−13^
l	Mean	−504.9050	−509.8908	−507.7595	−503.7786	−507.3658
	SD	0.0000	3.3030 × 10^−13^	2.1910 × 10^−13^	0.0000	6.5108 × 10^−13^
Behavior of failure intensity		Decreasing	Increasing	Increasing	Bathtub-shaped	Bathtub-shaped
Type of repair		Perfect	Imperfect	Minimal	Perfect	Minimal

**Table 6:** Mean absolute errors for the GRP models for data set #2; best value in bold.

	Weibull-GRP	q-Exponential-GRP	q-Weibull-GRP
MAE	15.2202 (A); 3.5504 (B)	2.5209	13.7140 (A); 2.1279 (B)

**Table 7:** Average time, in minutes, and average number of iterations for convergence (in parentheses) required by a PSO replication per GRP model and application example.

Application example	Weibull-GRP	q-Exponential-GRP	q-Weibull-GRP
1	0.0462 (881)	2.7446 (2057)	2.5520 (1514)
2	0.1009 (992)	3.9870 (1296)	42.2198 (3147)

**Table 8:** Average time, in seconds, required by the Monte Carlo simulation (10,000 replications) per GRP model and application example.

Application example	Weibull-GRP	q-Exponential-GRP	q-Weibull-GRP
1	1.0704	0.8385	1.1914
2	1.8857 (A); 1.6832 (B)	1.6626	2.2155 (A); 2.2607 (B)
